# Clinical Effect of Shenfu Injection in Patients with Septic Shock: A Meta-Analysis and Systematic Review

**DOI:** 10.1155/2015/863149

**Published:** 2015-06-14

**Authors:** Zijun Mou, Zhengtao Lv, Yi Li, Meng Wang, Qun Xu, Xuezhong Yu

**Affiliations:** ^1^Emergency Department, Peking Union Medical College Hospital, Beijing 100730, China; ^2^Department of Orthopedics, Tongji Hospital, Tongji Medical College, Huazhong University of Science and Technology, Wuhan 430030, China; ^3^Department of Epidemiology and Biostatistics, Institute of Basic Medical Sciences Chinese Academy of Medical Sciences, School of Basic Medicine Peking Union Medical College, Beijing 100005, China

## Abstract

*Purpose*. To conduct a meta-analysis evaluating the efficacy of Shenfu injection for treating patients with septic shock when compared with conventional therapy. *Methods*. Eight databases including Pubmed, EMBASE, Cochrane Library, ISI Web of Science, CNKI, Wanfang, VIP, and CBM were searched up to October 2014. Randomized controlled trials assessing the efficacy of Shenfu injection were identified. Mean arterial pressure, heart rate, lactate, and mortality were included as outcome measurements. *Results*. We analyzed data from 12 randomized controlled trials involving 904 participants. Compared with conventional therapy, Shenfu injection could further increase the mean arterial pressure at 1 hour (SMD 0.38; 95% CI, 0.01–0.74) and 6 hours (SMD 0.82; 95% CI, 0.03–1.61). Shenfu injection could further normalize heart rate at 6 hours (SMD −0.90; 95% CI, −1.47–0.33) and clear serum lactate at 6 hours (SMD −0.51; 95% CI, −0.70–0.32) and 24 hours (SMD, 0.52; 95% CI, −0.77–0.26). As the endpoint of mortality was not unified, it was not meta-analyzed. *Conclusions*. Based on the findings in present review, Shenfu injection is more effective than conventional therapy in increasing mean arterial pressure, normalizing heart rate, clearing serum lactate, and reducing mortality. These results should be confirmed in higher level clinical trials in the future.

## 1. Introduction

Septic shock is characterized by an alteration in tissue perfusion associated with persistent arterial hypotension [1]. It is generally defined as systolic arterial pressure of less than 90 mm Hg, despite adequate fluid resuscitation [2]. This leads to organ dysfunction and even death in around 50% of cases [3].

The fundamental principles for the management of sepsis include early recognition, control of the infection source, appropriate and timely administration of antimicrobial drugs, and resuscitation with intravenous fluids and vasoactive drugs [4]. Fluid resuscitation is essential for the restoration and maintenance of adequate intravascular volume and organ perfusion [5]. The goal of cardiovascular resuscitation of septic shock is to improve organ perfusion, often by increasing the mean arterial pressure (MAP) [6]. Recommendations suggest that MAP of around 65 mm Hg should initially be targeted, since MAP below this value may be associated with a worse evolution [7, 8].

Once fluid resuscitation is insufficient to restore hemodynamic stability, vasopressor therapy is typically required to restore organ perfusion. Although norepinephrine is the current recommended mainstay of sepsis treatment for hypotension, excessive adrenergic stress has multiple adverse effects including direct myocardial damage, insulin resistance, thrombogenicity, immunosuppression, and enhanced bacterial growth [9]. High plasma catecholamine levels, the extent and duration of catecholamine therapy, and tachycardia are all independently associated with poor outcomes in critically ill patients [10, 11].

Septic patients often have an elevated heart rate (HR), even after excluding common causes of tachycardia such as hypovolemia, anemia, pain, and agitation [11]. An elevated HR is associated with adverse outcomes in septic shock and thus represents the extent of disease severity [12]. Heart rate reduction considerably lowers cardiac energy demands, thereby creating a better balance between myocardial energy generation and expenditure in conditions of impaired energy production [13]. In addition, sepsis impairs the ability of tissues to extract oxygen so that adenosine triphosphate (ATP) generation from glucose oxidation is supplemented by ATP generation from glycolysis, leading to lactate production [14]. Presence of elevated lactate levels was associated with a significantly increased mortality in patients with vasopressor-dependent septic shock and this hyperlactatemia represents a persistent perfusion deficit [15]. Thus, elevated blood lactate should be a trigger for early escalation of care, including resuscitation, irrespective of arterial blood pressure [16, 17].

Shenfu injection is a well-known Chinese traditional medicine, which is made of Red Radix Ginseng and Radix* Aconitum carmichaelii*, with the active ingredients of ginseng saponin and aconitum alkaloids. Its clinical indications include Tuojue in the Chinese traditional medicine (meaning shock). Pharmacological studies have shown that it could elevate blood pressure and improve microcirculation against inflammatory reaction.

Shenfu injection as a common emergency medicine is frequently used in the national hospital emergency department, showing good curative effect in rescuing septic shock and resuscitation [18, 19]. However, there is little published information to warrant Shenfu injection as a standard treatment of septic shock. The aim of our current study is to evaluate the clinical effect of Shenfu injection when compared with conventional therapy based on randomized controlled trials (RCTs).

## 2. Material and Methods

### 2.1. Literature Search Strategy

A systematic literature search of Cochrane Library, EMBASE, ISI Web of Science, and PubMed was conducted. Chinese databases including China Knowledge Resource Integrated (CNKI) Database, Wanfang Database, VIP, and Chinese Biomedical Literature (CBM) Database were also scrutinized for the identification of trials. All the above databases were searched from their inception dates up to the latest issue (October 2014). No language restriction was used.

The following medical subject headings or keywords were used for English databases: Shenfu, septic shock, and toxic shock. For Chinese databases we used free text terms “shenfu” and “nong du xing xiu ke” or “gan ran xing xiu ke.” In addition, the bibliographies of relevant systematic reviews and clinical guidelines were manually searched. The reference section of each study was also searched.

### 2.2. Inclusion Criteria

Studies that met the following criteria were included: (a) the enrolled patients were diagnosed with septic shock, the age of enrolled patients was >16 years, no restrictions on race or sex were imposed, and the number of included patients in both groups had to be >10; (b) the included studies were required to be randomized controlled trials aiming to assess the efficacy of Shenfu injection for septic shock; (c) experimental groups mainly received Shenfu injection and conventional therapy, without differentiating the administration method of Shenfu injection; (d) patients in control groups were treated in conventional ways, which included anti-infection, fluid resuscitation, nutrition support, correct acidosis, and administration of dopamine, and the drugs administered in experimental groups had to be in accordance with the drugs utilized in control groups; (e) the outcome measurements had to include the MAP, HR, lactate, or mortality.

### 2.3. Exclusion Criteria

The three exclusion criteria for each identified study were as follows: (a) articles regarding animal experiments, review articles, case reports, or expert experience reports; (b) nonrandomized studies; (c) studies that were duplicates for retrieving or publishing.

### 2.4. Data Extraction

Two investigators (Zhengtao Lv and Zijun Mou) screened each article independently and each one was blinded to the findings of the other reviewer. In accordance with the predetermined inclusion and exclusion criteria, two reviewers independently performed a strict screening to identify qualified articles, and they extracted data independently from these eligible articles using a standardized collection form, which included first author, year of publication, study design, cohort sizes, baseline characteristics for participants in different groups, guidelines for management, intervention treatments, main outcome assessments, timing of outcome measure, and follow-up periods after treatments. If the required information was not available in the included studies, attempts were made to contact the authors of the original papers via e-mail. Any disagreement between reviewers was resolved through discussion until a consensus was reached. The third review author (Yi Li) was consulted in case a consensus could not be reached.

### 2.5. Quality Assessment

The Cochrane Collaboration's tool was used to assess the risk of bias in included studies, which was based on seven items: random sequence generation, allocation concealment, blinding of participants and personnel, blinding of outcome assessment, incomplete outcome data, selective reporting, and other sources of bias [20]. The response for each criterion was reported as low risk of bias, high risk of bias, and unclear risk of bias. Two reviewers evaluated the quality of trials independently.

### 2.6. Data Synthesis and Analysis

The meta-analysis and statistical analyses were performed by using RevMan 5.3 analyses software of the Cochrane Collaboration. Odds ratio (OR) and the associated 95% confidence intervals (CIs) were calculated for mortality. The standard mean difference (SMD) was calculated for MAP, HR, and serum lactate using the same methodology. Before the data of included studies was combined, heterogeneity between trial results was estimated using a standard chi-square test and the Higgins *I* ^2^ test (*P* > 0.1 and *I* ^2^ < 50% indicate acceptable heterogeneity). We pooled data across studies using random effect models if statistical heterogeneity existed; otherwise, a fixed effect model would be used. In case of heterogeneity, subgroup analysis was conducted. Publication bias was assessed via a funnel plot if the number of included studies is equal to or greater than 5.

## 3. Results

### 3.1. Literature Search Results

**Figure 1:**
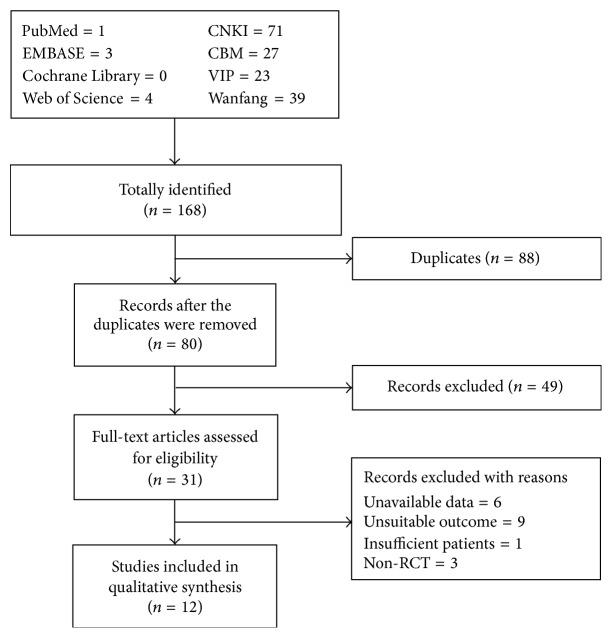
Flowchart of the literature search and study selection.

An initial search of RCTs yielded 168 potential literature citations, including 39 records from Wanfang, 71 from CNKI, 23 from VIP, 27 from CBM, 1 from PubMed, 0 from Cochrane Library, 3 from EMBASE, and 4 from ISI Web of Science. 88 studies were deleted because they were duplicates. According to the predetermined selection criteria, 31 potentially relevant studies were selected and retrieved for a full-text assessment after screening titles and abstracts. Of the remaining 31 articles, 6 studies were excluded because their data was unavailable, 9 studies were excluded because they employed unsuitable outcome, 1 study was excluded because the number of included patients was <10, and 3 studies were excluded because they were not RCTs. Finally, 12 studies met our inclusion criteria and were included in the meta-analysis. The literature screening process is summarized in a flowchart (Figure 1).

### 3.2. Study Characteristics

The main characteristics of the 12 trials are listed in Tables 1 and 2. These studies were all conducted by Chinese investigators and published between 2007 and 2014. Each study was performed at a single center. The 12 RCTs included a total of 904 patients with septic shock: 464 patients in the Shenfu injection group and 440 patients in the control group. Age of the participants ranged from 16 to 83 years. All 12 trials used 2-parallel-arm designs; Shenfu injection plus conventional therapy was compared with conventional therapy in these RCTs. Four studies followed the “surviving sepsis campaign guidelines for management of severe sepsis and septic shock” published in 2004 (SSC 2004) [21]; three studies followed the “surviving sepsis campaign: international guidelines for management of severe sepsis and septic shock” published in 2008 (SSC 2008) [7]; the guidelines followed by the other five studies were not reported. The timing of outcome measurements ranged from 1 hour to 28 days. Only one study [19] mentioned follow-up after treatment. Seven studies [18, 22–27] employed MAP as outcome measure, eight studies [18, 22–28] employed HR as outcome measure, eight studies [18, 22, 23, 25, 26, 29, 30] utilized lactate as outcome measure, and seven studies [18, 19, 23, 25, 27, 30, 31] employed mortality as outcome measure.

### 3.3. Risk of Bias

**Figure 2:**
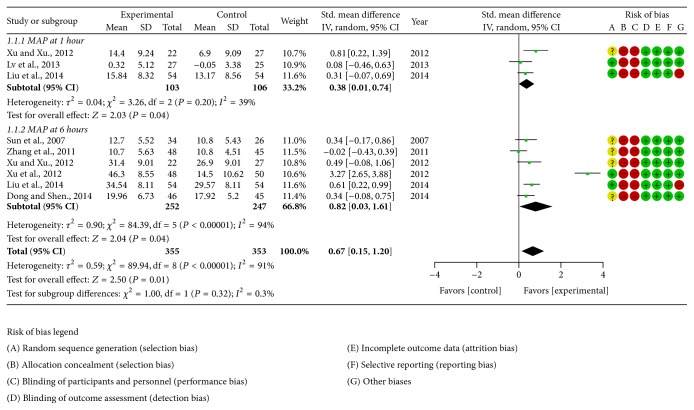
Forest plot of Shenfu injection plus conventional therapy versus conventional therapy: MAP; the authors' judgment about each risk of bias item for each included study.

**Figure 3:**
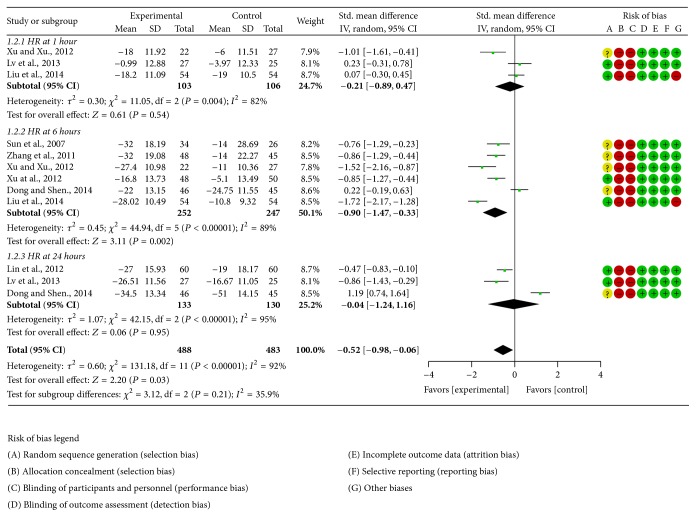
Forest plot of Shenfu injection plus conventional therapy versus conventional therapy: HR; the authors' judgment about each risk of bias item for each included study.

**Figure 4:**
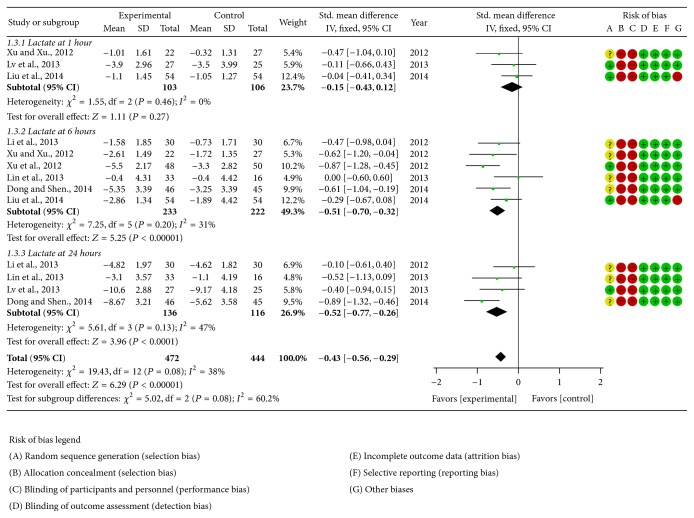
Forest plot of Shenfu injection plus conventional therapy versus conventional therapy: lactate; the authors' judgment about each risk of bias item for each included study.

The methodological quality of selected trials was assessed using the Cochrane Collaboration's tool. All of the studies included suggested randomization, but only five studies reported the method of random sequences generation. All studies failed to report details about allocation concealment. The blinding of outcome measurement was judged to low risk of bias because the outcomes were unlikely to be influenced by lack of blinding but there was high risk bias for blinding the participants or personnel in all studies. The number of dropouts and reasons for withdrawal were not reported in any of the above studies. When it comes to selective reporting bias, there was a low risk of bias since we only included the studies which used HR, MAP, lactate, or mortality as outcome measures. All studies had low risk of other biases except two studies that did not report baseline similarity. Finally, all studies were judged to be of a poor methodological quality. The judgment of risk of bias was presented in corresponding forest plots (Figures 2, 3, and 4).

### 3.4. Meta-Analyses Results

#### 3.4.1. MAP

Seven studies [18, 22–27] employed MAP as outcome measure; since there was obvious heterogeneity among included studies (*τ* ^2^ = 0.59; *χ* ^2^ = 89.94, degree of freedom (df) = 8 (*P* < 0.00001); *I* ^2^ = 91%), the random effect model was utilized for statistical analysis. The pooled MAP at 1 hour after treatment indicated that Shenfu injection further increased MAP when compared with conventional care (0.38 [0.01,0.74]); the pooled MAP at 6 hours after treatment indicated that Shenfu injection further increased MAP when compared with conventional care (0.82 [0.03,1.61]) (Figure 2).

#### 3.4.2. HR

Eight studies [18, 22–28] employed HR as outcome measure; since there was obvious heterogeneity among included studies (*τ* ^2^ = 0.60; *χ* ^2^ = 131.18, df = 11 (*P* < 0.00001); *I* ^2^ = 92%), the random effect model was utilized for statistical analysis. The pooled HR at 1 hour after treatment indicated that there was no significant difference between Shenfu injection and conventional care (−0.21 [−0.89,0.47]), the pooled HR at 6 hours after treatment indicated that Shenfu injection further reduced HR when compared with conventional care (−0.90 [−1.47, −0.33]), and the pooled HR at 24 hours after treatment indicated that there was no significant difference between Shenfu injection and conventional care (−0.04 [−1.24,1.16]) (Figure 3).

#### 3.4.3. Lactate

Eight studies [18, 22, 23, 25, 26, 29, 30] utilized lactate as outcome measure. Fixed effect model was used for statistical analysis because there was no obvious heterogeneity among studies. The pooled lactate at 1 hour after treatment indicated that there was no significant difference between Shenfu injection and conventional care (−0.15 [−0.43,0.12]), the pooled lactate at 6 hours after treatment indicated that Shenfu injection further cleared serum lactate when compared with conventional care (−0.51 [−0.70, −0.32]), and the pooled lactate at 24 hours after treatment indicated that Shenfu injection further reduced serum lactate when compared with conventional care (−0.52 [−0.77, −0.26]) (Figure 4).

#### 3.4.4. Mortality

Seven studies [18, 19, 23, 25, 27, 30, 31] employed mortality as outcome. As the endpoint of outcome measure was not unified, we only listed the mortality rate and the corresponding endpoint in Table 3. Except in Dong and Shen's study, a decreased trend of mortality and statistical difference could be detected in the other six studies.

### 3.5. Publication Bias

**Figure 5:**
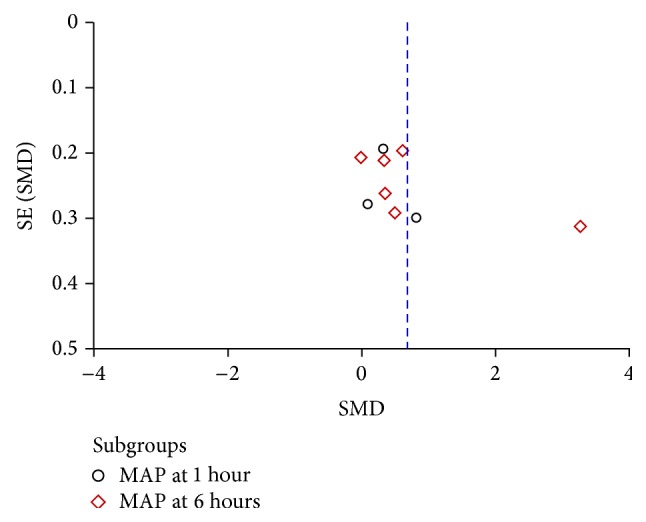
Funnel plot of Shenfu injection plus conventional therapy versus conventional therapy: MAP.

**Figure 6:**
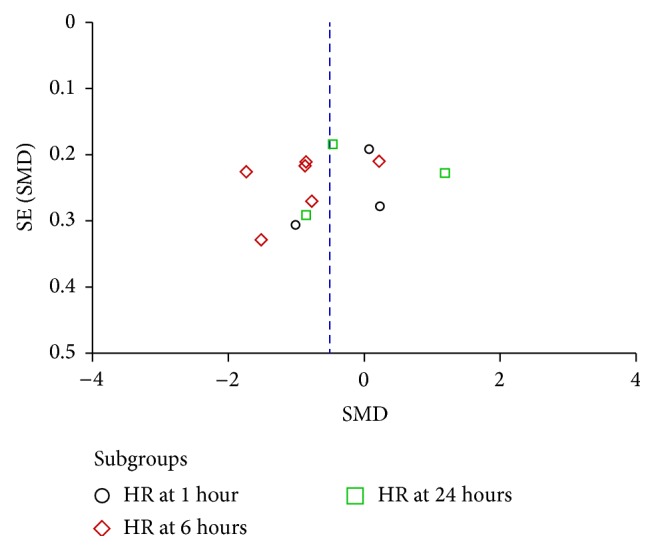
Funnel plot of Shenfu injection plus conventional therapy versus conventional therapy: HR.

**Figure 7:**
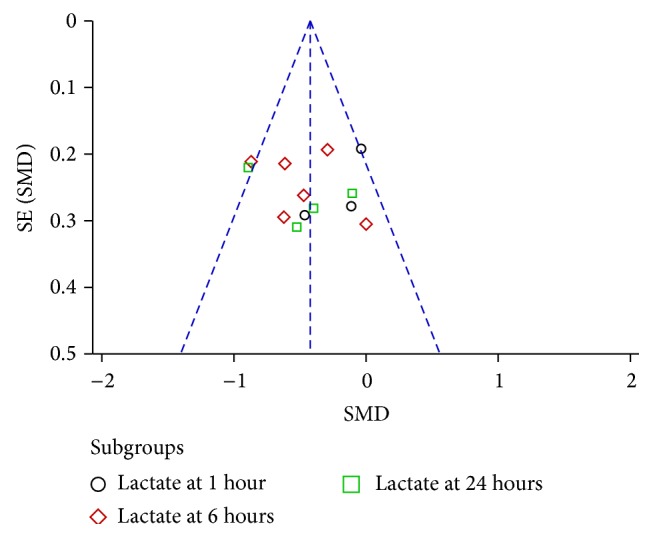
Funnel plot of Shenfu injection plus conventional therapy versus conventional therapy: lactate.

The publication bias was explored via funnel plots (Figures 5, 6, and 7). Points in Figures 5 and 6 presented asymmetry suggesting the possibility of publication bias. Points in Figure 7 seemed to be symmetric, which indicates no obvious publication bias. Given that all these selected articles were published in Chinese academic journals, the potential of publication bias could not be excluded.

## 4. Discussion

In summary, 12 RCTs including 904 patients were selected in our meta-analysis. Based on the findings of the present systematic review, Shenfu injection could further increase MAP, clear serum lactate, normalize HR, and reduce mortality when compared with conventional therapy. However, the potential beneficial effect from Shenfu injection is possibly overstated owing to the generally low methodological qualities of the included RCTs.

The overall methodological quality of our selected trials was judged to be poor, which might reduce the validity and value of the evidence examined in the present review. No studies achieved a low risk of bias as all studies had an unclear or high risk of bias within at least one major domain. Many studies failed to provide experimental methodologies in detail. All the included trials claimed randomization but part of them did not describe information about random sequence generation to estimate whether the randomization procedures had been carried out appropriately. No study employed the strategy of double-blinding, which might lead to the appearance of placebo effect and exaggeration of conclusions. Among the 12 included trials, no study reported number of dropouts and reasons for withdrawal. No study employed intention-to-treat analysis, so the conclusions regarding the clinical effect of Shenfu injection might be overstated.

In routine clinical practice, invasive methods such as thermodilution technique are not often indicated, and less invasive techniques, such as echocardiography and pulse contour methods, are not always available [32]. Other hemodynamic variables are frequently used, with satisfactory MAP often a key target, especially in hemodynamically unstable patients [33]. The lactate clearance has also been associated with decreased mortality in patients with severe sepsis and septic shock as well as with multiple organ dysfunction and systemic immunologic activation and inflammation [34]. Lactate clearance should be considered an additional goal of early sepsis resuscitation [35]. Consequently, we included these trials, which employed HR, MAP, serum lactate, and mortality as outcome.

Shenfu injection is an extract of traditional Chinese herbs, which mainly consists of ginsenoside and aconitine [36]. Modern pharmacological research shows that ginsenoside can improve ischemic myocardium metabolism, scavenge free radicals, protect myocardial ultrastructure, and reduce Ca^2+^ overload, and aconitine can enhance heart contractility, improve coronary circulation, and decrease the effect of acute myocardial ischemia [37]. In addition, aconitine contains noradrenaline salsolinol, which has excitatory effects on p receptors and *α*-adrenergic receptors, which can significantly increase cerebral blood flow by improving MAP [38]. Shenfu injection could also restore the ability of Na^+^-K^+^-ATPase and Ca^2+^-ATPase enzyme activities; this may be one of the mechanisms by which Shenfu injection could attenuate the myocardial dysfunction [39]. Moreover, Shenfu injection reduces the expression of TNF-*α* to block the vicious circle of inflammatory response, improve systemic microcirculation, and prolong the hypoxia tolerance duration [40]. The clinical effect of Shenfu injection was reflected in increase of MAP, reduction of HR, and clearance of serum lactate.

Subgroup analyses were conducted according to the timing of outcome measures. At 1 hour after Shenfu injection treatment, the pooled data revealed no significant difference of HR and serum lactate level between Shenfu group and control group, but the MAP was further increased in Shenfu groups. At 6 hours after treatment, a statistical difference could be detected in all three parameters, suggesting that Shenfu injection started to normalize HR, increase MAP, and help to clear serum lactate within 6 hours. And even at 24 hours after treatment, lactate level was further reduced in Shenfu injection group compared to control group. In terms of HR, there was no significant difference between Shenfu injection and conventional therapy at 24 hours after treatment. The findings of our work indicate that Shenfu injection helps to improve organ perfusion by increasing MAP, cut down the load of the heart, and reduce the oxygen consumption in early recovery of septic shock. In addition, the reduced blood lactate concentration suggests that Shenfu injection is capable of improving tissue hypoperfusion and the microcirculation. In a word, Shenfu injection was effective in the treatment of septic shock.

*Limitations*. In our present review, we included these studies which employed HR, MAP, serum lactate, and mortality as outcome. Changes of these parameters reflected the improvement of microcirculation and protective effect on myocardial cells of Shenfu injection. However, these adopted indicators are not comprehensive enough for the systematic assessment of Shenfu injection. Some well-established indicators such as APACHE/SAPS/SOFA scores, mechanical ventilation rates, renal replacement, or acute kidney injury rates were not reported by our selected trials.

Results of our systematic review were somewhat limited due to the poor methodological quality of included trials. Future randomized controlled trials should employ improved methodologies and reporting specifications as follows: (a) all clinical studies of Shenfu injection should be registered and comply with the Consolidated Standards of Reporting Trials (CONSORT) statement; (b) the sample sizes should be calculated before the start of the trials; (c) the generation of random allocation sequences and allocation concealment should be provided in detail; (d) these studies should be double-blinded and placebo-controlled; (e) the standard of diagnosis should be unified and widely accepted; (f) all adverse events associated with Shenfu injection should be reported and rigorously assessed.

## 5. Conclusion

Results of the present systematic review suggest that Shenfu injection could further increase MAP, normalize HR, clear blood lactate, and reduce mortality when compared with conventional therapy. However, the conclusion should be interpreted cautiously due to the poor methodological qualities of included studies. Additional RCTs with large-scale, rigorous study design and strict reporting specification are required.

## Figures and Tables

**Table 1:** Characteristics of included trials.

Authors, year	Nation	Study design	Sample size (*n*1/*n*2)	Age (mean or range)
Sun et al., 2007 [24]	China	RCT	60 (34/26)	E: 18–65; C: 18–60

Sun et al., 2008 [31]	China	RCT	60 (30/30)	E: 18–65; C: 18–60

Zhang et al., 2011 [27]	China	RCT	93 (48/45)	E: 16–72; C: 17–70

Li et al., 2013 [29]	China	RCT	60 (30/30)	E: 25–82; C: 22–79

Lin et al., 2012 [28]	China	RCT	120 (60/60)	E: 32–81; C: 31–83

Xu and Xu, 2012 [25]	China	RCT	49 (22/27)	E: 54 ± 0.3; C: 55 ± 0.2

Xu et al., 2013 [26]	China	RCT	98 (48/50)	E: 57 ± 7.0; C: 60 ± 8.0

He et al., 2013 [19]	China	RCT	64 (32/32)	Not reported

Lin et al., 2013 [30]	China	RCT	49 (33/16)	E: 79.8 ± 12.6; C: 76.5 ± 13.2

Lv et al., 2013 [23]	China	RCT	52 (27/25)	Not reported

Dong and Shen, 2014 [18]	China	RCT	91 (46/45)	E: 68.34; C: 69.56

Liu et al., 2014 [22]	China	RCT	108 (54/54)	Not reported

**Table 2:** Interventions and outcomes of included studies.

Authors, year	Guidelines for management	Experimental treatment	Control treatment	Timing of outcome measure	Follow-up	Main outcome
Sun et al., 2007 [24]	SSC 2004	Conventional therapy plus Shenfu injection (120 mL/day)	Conventional therapy (fluid resuscitation, dopamine)	6 hours	Not reported	HR, MAP
Sun et al., 2008 [31]	SSC 2004	Conventional therapy plus Shenfu injection (120 mL/day)	Conventional therapy (fluid resuscitation, dopamine)	14 days	Not reported	Mortality
Zhang et al., 2011 [27]	SSC 2004	Conventional therapy plus Shenfu injection (120 mL/day)	Conventional therapy (fluid resuscitation, dopamine, correct acidosis, anti-infection)	6 hours, 14 days	Not reported	HR, MAP, mortality
Li et al., 2013 [29]	SSC 2008	Conventional therapy plus Shenfu injection (100 mL/day)	Conventional therapy (fluid resuscitation, dopamine, correct acidosis, anti-infection)	6 hours, 12 hours, 24 hours	Not reported	Lactate
Lin et al., 2012 [28]	Not reported	Conventional therapy plus Shenfu injection (100 mL/day)	Conventional therapy (fluid resuscitation, dopamine, correct acidosis, anti-infection)	24 hours	Not reported	HR
Xu and Xu, 2012 [25]	Not reported	Conventional therapy plus Shenfu injection (50 mL + 15 mL/hour)	Conventional therapy (fluid resuscitation, dopamine, correct acidosis, anti-infection)	1 hour, 2 hours, 3 hours, 4 hours, 5 hours, 6 hours	Not reported	Lactate, MAP, HR
Xu et al., 2013 [26]	SSC 2004	Conventional therapy plus Shenfu injection (100 mL/day)	Conventional therapy (fluid resuscitation, dopamine, correct acidosis, anti-infection)	6 hours	Not reported	Mortality, MAP, HR, lactate
He et al., 2013 [19]	SSC 2008	Conventional therapy plus Shenfu injection (100 mL/day)	Conventional therapy (anti-infection treatment, fluid resuscitation, glucose monitoring, cortical hormone)	15 days	6 months	Lactate, mortality
Lin et al., 2013 [30]	Not reported	Conventional therapy plus Shenfu injection (120 mL/day)	Conventional therapy (treatment of primary condition, fluid resuscitation, anti-infection, nutrition support)	6 hours, 24 hours, 72 hours	Not reported	Lactate, mortality
Lv et al., 2013 [23]	Not reported	Conventional therapy plus Shenfu injection (1 mL/Kg)	Conventional therapy (fluid resuscitation, dopamine, correct acidosis, anti-infection)	1 hour, 8 hours, 12 hours, 24 hours	Not reported	HR, MAP, lactate, mortality
Dong and Shen, 2014 [18]	SSC 2008	Conventional therapy plus Shenfu injection (100 mL/day)	Conventional therapy (fluid resuscitation, dopamine, correct acidosis, anti-infection)	6 hours, 24 hours, 28 days	Not reported	HR, MAP, lactate, mortality
Liu et al., 2014 [22]	Not reported	Conventional therapy plus Shenfu injection (150 mL/day)	Conventional therapy (fluid resuscitation, norepinephrine)	1 hour, 2 hours, 3 hours, 4 hours, 5 hours, 6 hours	Not reported	MAP, HR, lactate

*Note*. SSC 2004: “surviving sepsis campaign guidelines for management of severe sepsis and septic shock” published in 2004; SSC 2008: “surviving sepsis campaign: international guidelines for management of severe sepsis and septic shock” published in 2008; 50 mL + 15 mL/hour: intravenous administration of 50 mL Shenfu injection prior to 15 mL/hour continuous infusion, and the total dose of Shenfu injection a day is 150 mL.

**Table 3:** Mortality of experimental and control groups and the associated *P* value.

Authors, year	Endpoint	Experimental group	Control group	*P* value
Sun et al., 2008 [31]	14 days	6.67% (2/30)	13.33% (4/30)	*P* < 0.05
Zhang et al., 2011 [27]	14 days	4.17% (2/48)	15.56% (7/45)	*P* < 0.05
Xu et al., 2013 [26]	72 hours	12.50% (6/48)	26.00% (13/50)	*P* < 0.05
He et al., 2013 [19]	28 days	3.13% (1/32)	9.38% (3/32)	*P* < 0.01
Lin et al., 2013 [30]	24 hours	9.09% (3/33)	37.50% (6/16)	*P* < 0.05
Lv et al., 2013 [23]	24 hours	7.40% (2/27)	24.00% (6/25)	*P* < 0.05
Dong and Shen, 2014 [18]	28 days	56.52% (26/46)	64.44% (29/45)	*P* > 0.05
